# Assessment of blood pressure control in adult hypertensive patients in eastern Sudan

**DOI:** 10.1186/s12872-018-0769-5

**Published:** 2018-02-07

**Authors:** Saeed M. Omar, Osama Elnour, Gamal K. Adam, Osman E. Osman, Ishag Adam

**Affiliations:** 1 Faculty of Medicine, Gadarif University, Gadarif, Sudan; 2 Faculty of Medicine, Omdurman University, Omdurman, Sudan; 3 Faculty of Medicine, Alneelain University, Khartoum, Sudan; 4 Faculty of Medicine, University of Khartoum, P.O. Box 102, Khartoum, Sudan

**Keywords:** Hypertension, Controlled blood pressure, Dyslipidemia, Adherence, Sudan

## Abstract

**Background:**

The rate of blood pressure (BP) control in adult hypertensive patients is poor and the reasons for poor control of BP pressure are not fully understood globally. This study aimed to assess the rate and factors associated with BP control in adult hypertensive patients in Sudan.

**Methods:**

A hospital-based cross-sectional study was conducted in adult hypertensive Sudanese patients at Gadarif Hospital in eastern Sudan from November 2016 to March 2017. Information on sociodemographic characteristics of the participants, comorbidities, antihypertensive medication, and adherence to antihypertensive medication was gathered from patients using a questionnaire. Fasting cholesterol and triglyceride levels were measured.

**Results:**

A total of 380 patients were enrolled. Of them, 234 (61.6%) were women. The mean (SD) age of the participants was 57.8 (11.1) years (range: 25–93 years). Over one-third (*n* = 147, 38.7%) of the participants were taking more than one antihypertensive medication. Approximately one-third (29.5%) of the participants were non-adherent to medication. The rate of BP control was 45.3%. In binary logistic regression analyses, age, sex, physical inactivity, adding salt to food, drinking coffee, body mass index, and the lipid profile were not associated with uncontrolled BP. However, non-adherence to medication was the main factor associated with uncontrolled BP (odds ratio = 5.29, 95% confidence interval = 3.16–8.83, *P* <  0.001).

**Conclusions:**

Almost half of hypertensive patients in follow-up have uncontrolled BP, mainly due to non-adherence to medicine. We recommend further research on drug adherence to improve the rate of BP control in this setting (Gadarif) of the Sudan.

**Electronic supplementary material:**

The online version of this article (10.1186/s12872-018-0769-5) contains supplementary material, which is available to authorized users.

## Background

Hypertension is estimated to increase from approximately 1.0 billion in 2000 to 1.5 billion by 2025 [1]. Unfortunately, the majority of patients’ blood pressure (BP) is poorly controlled in all settings, especially in countries with minimal resources. Uncontrolled BP is defined as BP measurement of ≥140/90 mmHg. Uncontrolled BP is a major health problem and can lead to high morbidity and mortality risks, such as heart failure, coronary heart disease, stroke, and renal insufficiency [2, 3]. Recent publications have reported various rates and determinants for uncontrolled hypertension (e.g., age, sex, education) [4–8].

Among the known predictors of uncontrolled BP is non-adherence to antihypertensive medications and this can potentiate development of hypertensive complications [3, 9]. Adherence to antihypertensives is defined if the patients take at least 80% of their medications on a daily basis [10]. Despite the availability of effective medications, control of high BP is well below the expected level. Many factors have been reported to affect the level of adherence to antihypertensives. These factors include the level of education, access to medications, and the number of antihypertensive drugs used by the patients and their adverse effects [10, 11].

Hypertension is large health problem in Sudan where 35.7% of adults in North Sudan are hypertensive [12]. However, there are few published data on hypertension in Sudan [13, 14]. Research on hypertension is important for researchers, clinicians, and health planners. The current study was conducted to examine the rate and associated factors of uncontrolled BP at Gadarif Hospital in eastern Sudan.

## Methods

A cross-sectional study was conducted at Gadarif hospital from November 2016 to March 2017. After explaining the purpose of the study and signing informed consent, adult (≥ 20 years) hypertensive Sudanese patients who visited for follow-up were enrolled. Pregnant women and patients with poor cognitive functions were excluded. Sociodemographic characteristics e.g. age, sex, residence, education, health insurance, marital status, smoking (smokers were subject who smoked more than 100 cigarettes in their lives and reported any past-year smoking), alcohol consumption (one or more drink in the past month), duration of hypertension, and comorbidities (diabetes, thyroid, hyperlipidemia, and stroke) were gathered through a questionnaire which we developed it for this study (Additional file 1).

The participants were classified as physically active if they were being regularly involved in moderate or strenuous leisure activity for 4 h or more per week; otherwise, they were classified as physically inactive. BP (on the index day of the visit) was measured twice after resting for at least 10 min using an OMRON 3 (with an appropriate-size cuff) automated blood measuring device. The mean of two (at an interval of 1–2 min) blood pressure readings was calculated. The mean of the two readings was recorded. If the difference between the two readings was > 5 mmHg, re -measurements were taken until the stability of the reading was reached. The patient’s arm was kept/maintained at the level of the heart. Drug adherence was assessed by the Morisky score [15].

The patients’ weight and height were measured using standard procedures and body mass index (BMI) was computed as weight/height (m^2^). Cholesterol and triglyceride levels were measured using enzymatic methods.

The sample size of 380 subjects was determined. This sample size was based on previous studies where 40% of patients had uncontrolled BP [5] to detect a difference of 5% at α = 0.05 with a power of 80%. We assumed that 10% of the participants might not respond or have incomplete data.

A pilot study enrolling 20 participants was conducted, and changes were made in the questionnaires accordingly. The medical officers who collected the data were trained in data collection methods to standardize the data collection procedure to maintain data quality.

## Definitions

Hypertension was defined as sustained high blood pressure (systolic BP ≥140 or diastolic BP ≥90 mmHg) or reported regular use of antihypertensive medication [16]. Uncontrolled BP was defined as systolic BP of ≥140 mmHg and/or diastolic BP of ≥90 mmHg [17]. Controlled blood pressure was defined as systolic BP of < 140 mmHg and/or diastolic BP of < 90 mmHg. Morisky’s medication adherence scale (MMAS-8) was used to assess the adherence of antihypertensive among participants who were on treatment. MMAS-8 Scale contained four questions relating to use or forget to take the antihypertensive/medicine (two questions) and self-adjustment of medicine dosages (two questions). The questions have the options “Yes,” “No” to reply. The “Yes” and “No” rated answers were assigned a value of 1, and 0, respectively. Then, the aggregate number was used and categorized as 0 (high adherence), 1 to 2 (medium adherence), and non-adherence was considered if the score was ≥3 [15].

### Statistical analysis

Data were entered into a computer using SPSS for Windows (version 20.0). The chi-square test was used to compare proportions between patients with controlled and uncontrolled BP. The Kolmogorov–Smirnov test was used for testing the normality of continuous data (age, duration of hypertension, BMI, and lipid profile). The Student’s t-test and Mann–Whitney test were used to compare continuous parametric and non-parametric data, respectively, between the two groups (controlled and uncontrolled BP). Binary regression analyses were performed with uncontrolled BP as the dependent variable. Independent variables (age, sex, marital status, education, presence of comorbidity, alcohol intake, coffee intake, physical activity, measuring blood pressure at home, having medical insurance, BMI, and the lipid profile) were entered into the model if their univariate P was < 0.20. Odds ratios (ORs) and 95% confidence intervals (CIs) were calculated and a *P* value of < 0.05 was considered significant.

## Results

A total of 380 adult hypertensive patients were enrolled in the study. Among them, 234 (61.6%) patients were women. The mean (SD) age of the participants was 57.8 (11.1) years (range: 25–93 years). The majority (82.6) of the participants were married. Approximately two-fifths (44.7%) of the participants ingested salt with food. Forty-two (11.1%) participants were cigarette smokers and 12 (3.2%) were alcohol drinkers. The majority (58.2%) of the participants were drinking coffee. A total of 270 (71.8) participants had medical insurance (Table 1). The mean (SD) duration of hypertension was 6.7 (5.8) years. Half of the patients (50.3%) had hypertension for ≥5 years.

**Table 1:** The clinical and biochemical characteristics in adult hypertensive patient, eastern Sudan

Variable	Value	Percentage
Age (years)^a^	57.8	11.1
Male sex	146	38.4
Education ≤ secondary level	361	95.0
Married	314	82.6
Duration of hypertension, years^†^	5.0	53.0–8.0
Presence of comorbidity	184	48.4
Smoking/ex-smoking	42	11.1
Alcohol intake	12	3.2
Drinking coffee	220	58.0
Physically active	16	4.2
Adherence to drugs	268	70.5
Measuring blood pressure at home	22	5.8
Have medical insurance	273	71.8
Taking traditional medicine	24	24 (6.3)
Body mass index, kg/m^2b^	26.2	23.4–28.4
Cholesterol, mg/dl^b^	179.7	156.0–210.0
Triglycerides, mg/dl^b^	146.0	115.0–190.0

^a^Mean (SD), ^b^median (interquartile range)

**Fig. 1:**
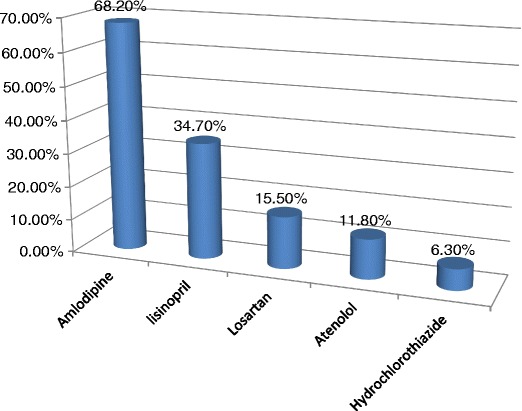
Antihypertensive drugs used by the patients in eastern Sudan

Over one-third (*n* = 147, 38.7%) of the participants were taking more than one antihypertensive medication. Approximately two-thirds (*n* = 259, 68.2%) of the participants were using calcium channel blockers (amlodipine). A total of 132 (34.7%), 59 (15.5%), 45 (11.8%), and 24 (6.3%) of the participants were using angiotensin II receptor blockers (lisinopril), angiotensin-converting enzyme inhibitors (losartan), beta-blockers (atenolol), and diuretics (hydrochlorothiazide), respectively (Fig. 1). The most common comorbidities were diabetes (*n* = 124, 32.6%), thyroid disease (*n* = 19, 5.0%), previous ischemic disease (*n* = 17, 4.5%), previous stroke (*n* = 15, 3.9%), heart failure (*n* = 4, 1.1%), and renal disease (*n* = 5, 1.3%). The mean (SD) systolic and diastolic blood pressure was 134.3 (15.8) mm Hg and 89.3 (46.3) mm Hg, respectively.

Approximately one-third (29.5%) of the participants were non-adherent to medication. The rate of BP control was 45.3%. There were no significant differences in age, sex, education, duration of hypertension, presence of comorbidity, drinking coffee, and alcohol intake between participants with controlled BP and those with uncontrolled BP. A significantly higher number of participants with uncontrolled BP were non-adherent to medicine and had higher cholesterol and triglyceride levels compared with those with controlled BP (all *P* <  0.001, Table 2).

**Table 2:** Comparison of clinical and biochemical characteristics between patients with controlled and uncontrolled blood pressure

Variable	Controlled blood pressure (*n* = 208)	Uncontrolled blood pressure (*n* = 172)	OR	95% CI	P
Age, years	58.1(10.9)	57.4 (11.4)	0.94	0.97–1.01	0.533
Male sex	74 (35.6)	72 (41.9)	0.76	0.50–1.16	0.244
Education ≤ secondary level	202 (97.1)	159 (92.4)	0.36	0.13–0.97	0.056
Married	169 (81.2)	145 (84.3)	0.90	0.60–1.36	0.647
Duration of hypertension, years	5.5(2.0–8.0)	5.0 (3.0–8.0)	1.01	0.97–1.04	0.523
Presence of comorbidity	103 (49.5)	81 (47.1)	0.90	0.60–1.36	0.680
Smoking/ex-smoking	17 (8.2)	25 (14.5)	1.91	0.99–3.67	0.070
Alcohol intake	4 (1.9)	8 (4.7)	2.48	0.73–8.40	0.150
Drinking coffee	119 (57.2)	101 (59.1)	1.07	0.71–1.62	0.754
Physically active	8 (3.8)	8(4.7)	1.22	0.44–3.32	0.799
Adherence to drugs	179 (86.1)	89 (51.7)	0.17	0.10–0.28	< 0.001
Measuring blood pressure at home	13 (6.2)	9 (5.2)	0.82	0.34–1.98	0.826
Have medical insurance	150 (72.1)	123 (71.8)	0.97	0.62–1.52	0.909
Taking traditional medicine	13 (6.3)	11 (6.4)	1.02	0.44–2.34	1.000
Body mass index, kg/m^2^	26.2 (23.1–28.0)	26.2 (23.7–29.0)	1.02	0.98–1.06	0.282
Cholesterol, mg/dl	168.0(142.2–194.2)	190.0 (160.0–210.0)	1.01	1.01–1.03	0.001
Triglycerides, mg/dl	130.0(111.2–167.0)	158.4 (124.2–195.0)	1.01	10.1–1.07	0.001

Values are mean (SD) or median (interquartile range)

In binary logistic regression analyses, age, sex physical inactivity, adding salt to food, drinking coffee, BMI, and the lipid profile were not associated with uncontrolled BP. However, non-adherence to medication was associated with uncontrolled BP (OR = 5.29, 95% CI = 3.16–8.83, *P* < 0.001, Table 3).

**Table 3:** Binary regression analyses of factors related to uncontrolled blood pressure in eastern Sudan

Variable	OR	95% CI	*P*
Male sex	0.87	0.53–1.43	0.594
Education ≤ secondary level	0.38	0.12–1.16	0.091
Smoking/ex-smoking	1.79	0.85–3.79	0.123
Alcohol intake	1.03	0.28–3.83	0.955
Non-adherence to drugs	5.29	3.16–8.83	< 0.001
Cholesterol, mg/dl	1.01	0.99–1.01	0.161
Triglycerides, mg/dl	1.01	0.99–1.01	0.750

## Discussion

The main findings of the current study were that the rate of uncontrolled BP was 45.3% and non-adherence to medication was the only factor associated with uncontrolled BP. In neighboring Ethiopia, Asgedom et al. reported that the rate of BP control was 50.3% and more than one-third (39.5%) of the participants were non-adherent to their medication [6]. Generally, our finding of the rate of BP control (54.7%) is similar to that in other African countries. The rate of BP control is 46.6%, 47.7%, and 41.9% in Ethiopia, Tanzania, and South Africa, respectively [18–20]. However, much lower BP control (24.6%) was reported in Cameroon [21]. The reason for this lower rate of BP control in Cameroon could be because a population-based study was performed, while facility-based findings were reported in other studies.

In the current study, there was no association between sociodemographic characteristics (including age, sex, and alcohol) and uncontrolled BP. These findings are similar to those observed in Tanzania, Cameron, and Nepal [7, 8, 19]. These findings are in contrast to those in a study conducted in Ethiopia in which age, physical inactivity, chat chewing, adding salt to food, and drinking coffee were significantly associated with uncontrolled BP [6]. In our study, education and health insurance status were not significantly associated with BP control. These findings are similar to those reported in Tanzania and Cameroon [8, 19]. However, in the United States, a previous study showed that among patients who were treated for hypertension, uninsured individuals were at higher risk of uncontrolled BP [22]. Notably, the rate (71.8%) of medical insurance in this setting of eastern Sudan was lower than that (87.5%) of medical insurance that was recently observed in Cameroon [8].

Our study showed a significant association of uncontrolled BP with non-adherence to antihypertensive medication, where non-adherent participants had a 5.29 times higher risk of uncontrolled BP. This finding is consistent with previous studies in various settings [6, 8, 19]. Our finding that 29.5% of the participants were non-adherent to medication is lower than previously observed in Kassala in eastern Sudan [14]. This previous study showed that 59.6% of patients were compliant to their medication as assessed by a different method (pill count method). Recently, Asgedom et al. reported that 39.5% of hypertensive Ethiopian patients were not adherent to medicine [6]. Low adherence to antihypertensive treatment is associated with a significant increase in the risk of cardiovascular events and a higher rate of hospital admission [23, 24]. Therefore, adherence to antihypertensive medicine in this setting should be encouraged. Previous studies have shown that participants were not adherence and intentionally avoided antihypertensive because of; long term medication and fearing side effects [7], complexity of the regimen used in the the treatment of asymptomatic disease (hypertension) [25].Various aspects of the new guidelines aimed to assist researchers working on hypertension in the term of; lifestyle; nutrition, new drugs and adopting effective drug delivery systems have been recently reviewed and these might improve to control hypertension and reducing its complications [26].

### Limitations

There are some limitations of this study. One of the limitations of the current study was that it was a facility-based study. The actual rate of uncontrolled BP might have been underestimated in this study because it might not have reflected BP at the community level.

## Conclusions

Almost half of the hypertensive patients at follow-up in a hospital in eastern Sudan have uncontrolled BP, mainly due to non-adherence to medicine. We recommend better health education and research on drug adherence to improve the rate of BP control in this setting.

## Additional file

Additional file 1: Questionnaire for the assessment of blood pressure control in adult hypertensive patients in eastern Sudan. (PDF 338 kb)

## Abbreviations

BMI: Body mass index
BP: Blood pressure
CI: Confidence interval
ORs: Odds ratios
SD: Standard deviation
