# Supplementary material for: Assessment of blood pressure control in adult hypertensive patients in eastern Sudan
Source: BMC Cardiovasc Disord. 2018 Feb 7;18:26. doi: 10.1186/s12872-018-0769-5 (PMC5803924; doi:10.1186/s12872-018-0769-5)
Supplement: Additional file 1: — Questionnaire for the assessment of blood pressure control in adult hypertensive patients in eastern Sudan. (PDF 338 kb) [file 12872_2018_769_MOESM1_ESM.pdf]

Questionnaire for the assessment of blood pressure control in adult hypertensive patients in eastern Sudan

Serial number------

Age-☐-

Gender male ☐ female ☐

Residence rural ☐ urban ☐

Marital status marrie☐ -unmarried-☐

Education: Nill/traditional☐ secondary☐ university and above☐

Health insurance Yes ☐ No ☐

Duration of HT☐ Number of drugs☐ Number of antihypertensive ☐

Other diseases: No ☐ Yes ☐

If Yes DM ☐ Thyroid ☐ Ischemic attach ☐ Heart failure ☐ Renal dis ☐

	**No**	**Yes**
Smoking	☐	☐
Alcoholic	☐	☐
Add salt	☐	☐
Drink coffee	☐	☐

Weight-☐--height-☐

Drug adherence ☐--Please see annex

Blood pressure, systolic-☐ diastoli☐---

Traditional medicine No- ☐ -Yes ☐ Using antilipids No ☐ Yes ☐

Home measurement No ☐ Yes ☐

Antihypertensive:

☐ Calcium channel blockers (amlodipine)

☐ Angiotensin II receptor blockers (lisinopril)

☐ Angiotensin-converting enzyme inhibitors (losartan)

☐ Beta-blockers (atenolol)

☐ Diuretics (hydrochlorothiazide)

Lipids cholesterols ------triglycerides---- HDL LDL
